# Outcomes and potential mechanism of a protocol to optimize foot orthoses in patients with rheumatoid arthritis

**DOI:** 10.1186/s12891-020-03364-5

**Published:** 2020-06-04

**Authors:** Marloes Tenten-Diepenmaat, Joost Dekker, Jos W. R. Twisk, Elleke Huijbrechts, Leo D. Roorda, Marike van der Leeden

**Affiliations:** 1 Amsterdam Rehabilitation Research Center | Reade, Amsterdam, the Netherlands; 2 Department of Rehabilitation Medicine, Amsterdam UMC, Vrije Universiteit Amsterdam, Amsterdam, the Netherlands; 3 Amsterdam Public Health research institute, Amsterdam UMC, Amsterdam, the Netherlands; 4 Department of Epidemiology and Biostatistics, Amsterdam Public Health research institute, Amsterdam UMC, Amsterdam, the Netherlands; 5 Department of allied health professionals | Fontys Paramedische Hogeschool, Fontys University of applied sciences, Eindhoven, The Netherlands

**Keywords:** Rheumatoid arthritis, Foot, Orthoses, Orthotic device, Outcome assessment (health care), In-shoe plantar pressure measurements

## Abstract

**Background:**

Foot problems are highly prevalent in patients with rheumatoid arthritis. Treatment of foot problems related to rheumatoid arthritis often consists of custom made foot orthoses. One of the assumed working mechanisms of foot orthoses is redistribution of plantar pressure by creating a larger weight bearing area. Overall, the reported treatment effect of foot orthoses on foot pain in rheumatoid arthritis is small to medium. Therefore, we developed a foot orthoses optimization protocol for evaluation and adaptation of foot orthoses by using the feedback of in-shoe plantar pressure measurements. The objectives of the present study were: 1) to evaluate the 3-months outcomes of foot orthoses developed according to the protocol on pain, physical functioning and forefoot plantar pressure in patients with foot problems related to rheumatoid arthritis, and 2) to determine the relationship between change in forefoot plantar pressure and change in pain and physical functioning.

**Methods:**

Forty-five patients with foot problems related to rheumatoid arthritis were included and received foot orthoses developed according to the protocol. Outcome measures were assessed at baseline and after three months of wearing foot orthoses in 38 patients. Change scores and effect sizes (ES) were calculated for pain, physical functioning and plantar pressure. In a subgroup of patients with combined forefoot pain and high plantar pressure, the relationship between change in plantar pressure and change in pain and physical functioning was analyzed.

**Results:**

In the total group of 38 patients, statistically significant changes in pain (ES 0.69), physical functioning (ES 0.82) and forefoot plantar pressure (ES 0.35) were found. In the subgroup (*n* = 23) no statistically significant relationships were found between change in plantar pressure and change in pain or physical functioning.

**Conclusion:**

Foot orthoses developed according to a protocol for improving the plantar pressure redistribution properties lead to medium to large improvements in pain and physical functioning. The hypothesis that more pressure reduction would lead to better clinical outcomes could not be proven.

## Background

Foot problems are highly prevalent in patients with rheumatoid arthritis (RA) [1–5]. Inflammation and synovitis of foot joints may lead to changes in foot structure and foot function [1, 6]. Abnormal foot function can result in high plantar foot pressures and subsequent foot pain and disability [7, 8]. This process mainly affects the forefoot [1, 7]. Previous research showed that RA patients with foot problems experience limitations in daily activities and a reduced quality of life [9, 10].

Treatment of RA-related foot problems often consists of custom made foot orthoses (FOs) and a shoe advice by a podiatrist, especially in the early stage of the disease [11]. One of the assumed working mechanisms of FOs is redistribution of plantar pressure by creating a larger weight bearing area [12–14]. Overall, the reported treatment effect of FOs on foot pain in RA is small (effect size 0.4) [15], to medium (effect size 0.45) [16]. The immediate feedback from in-shoe plantar pressure measurements seems promising in optimizing the treatment effect of FOs, as shown in treatment with therapeutic footwear (including FOs) in diabetic foot patients [17]. Since the evaluation and subsequent adaptations of FOs in patients with RA is usually based on the patient’s feedback, a protocol for the use of in-shoe plantar pressure measurements was developed, based on the protocol previously tested by Bus et al. [17, 18]. The protocol included: (1) setting individual treatment goals on plantar pressure redistribution, (2) manufacturing custom-made FOs according to the patient’s needs, based on the clinical reasoning process of the podiatrist, and (3) evaluating and, if necessary, adapting FOs according to the feedback of in-shoe plantar pressure measurements (in one to three rounds). The adapted FOs showed, in a repeated single session design, small additional forefoot plantar pressure reduction over usual care FOs [18]. The immediate feedback of in-shoe plantar pressure measurements provided guidance in the clinical reasoning process of the podiatrist. The outcomes of FOs developed according to the FOs optimization protocol after 3 months follow-up on pain, physical functioning and forefoot plantar pressure are not yet known.

Since high plantar pressures are related to foot pain in RA it is hypothesized that a reduction of forefoot plantar pressure leads to reduction of pain and subsequent disability [7]. Nevertheless, there is a lack of evidence supporting this hypothesis. Previously published systematic reviews indicate that custom made FOs are effective in reducing forefoot plantar pressures [16] and pain in RA [15, 16]. However, the relationship between change in forefoot plantar pressure and change in pain has never been investigated. Furthermore, in our previous study investigating the FOs optimization protocol we found that a subgroup of patients with forefoot pain also had high forefoot plantar pressure at baseline [18]. This implies that only in the patients with combined forefoot pain and high forefoot plantar pressure, the mode of action of FOs may be related to plantar pressure reduction. Therefore, subgroup analysis is necessary to investigate whether pressure reduction is associated with outcomes on pain and physical functioning.

The objective of the present study was twofold: 1) to evaluate the outcomes of FOs developed according to the FOs optimization protocol on pain, physical functioning and forefoot plantar pressure in patients with RA-related foot problems, and 2) to determine the relationship between change in forefoot plantar pressure and change in pain and physical functioning.

## Methods

### Design

Patients of an outpatient center for rehabilitation and rheumatology in the Netherlands served as the study population for this quasi-experimental clinical trial. In a previously published proof of concept study the outcomes of FOs (developed by using a FOs optimization protocol) on immediate plantar pressure redistribution and the feasibility of the protocol were reported [18]. In this FOs optimization protocol, the feedback of in-shoe plantar pressure measurements was used for the evaluation and adaptation of FOs. For the purpose of the present study, in-shoe plantar pressure measurements were assessed (in the patient’s own shoes) without FOs at baseline (T0) and with FOs at 3 months after delivery (follow-up (T1)). Pain and physical functioning were measured before FOs delivery (T0) and after 3 months of wearing FOs (T1). The outcomes on pain, physical functioning and forefoot plantar pressure were analyzed in all included patients (total group). Out of this total group, a subgroup was selected of patients with combined forefoot pain and high plantar pressure (Peak Pressure ≥ 200 kPa in the central forefoot region (metatarsophalangeal joints 2–3) [17]) at baseline. We hypothesized that in these patients the working mechanism of FOs on pain and physical functioning outcomes is related to plantar pressure reduction. Therefore, the relationship between change in forefoot plantar pressure and change in pain and physical functioning was investigated in the subgroup. In addition, clinical characteristics were assessed. The medical ethics committee of the Slotervaart Hospital/Reade in Amsterdam approved this study (NL41146.048.12) and written informed consent was obtained from each patient.

### Patients

Consecutive patients, who were referred by a rheumatologist for podiatric treatment in a specialized center for rheumatology and rehabilitation, were approached to participate in the present study. Inclusion criteria were: 1) ≥18 years of age, 2) RA diagnosed by a rheumatologist according to the revised criteria of the American Rheumatism Association [19], 3) referral for podiatric treatment because of RA-related foot problems, and 4) indication for FOs according to the podiatrist. Exclusion criteria were: 1) another medical condition that underlies the foot problems, 2) not able to walk independently without using aids, and 3) inability to fill out questionnaires because of language or cognitive difficulties.

### Podiatric treatment according to the FOs optimization protocol

The podiatric treatment consisted of custom-made FOs (for both feet) according to the patient’s needs, based on the feedback of in-shoe plantar pressure measurements and the clinical reasoning process of the podiatrist [18]. If necessary, an individual advice (oral and written) concerning over-the-counter shoes was provided to the patient. The podiatric treatment was performed by three qualified podiatrists, accustomed to treating RA-related foot problems with 1.5, 5 and 11 years of experience.

**Fig. 1:**
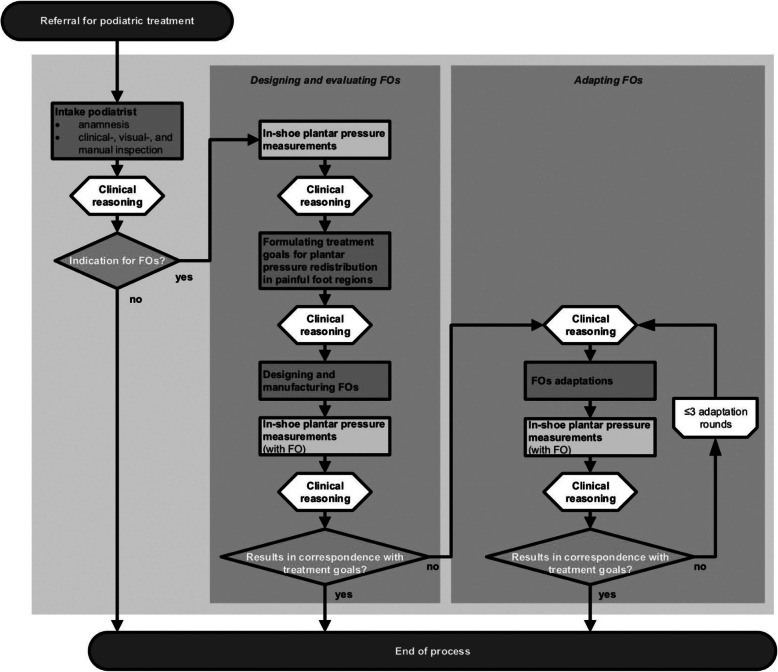
FOs optimization protocol

The process for designing, evaluating and adapting FOs according to the FOs optimization protocol is shown in Fig. 1. This process started with a podiatric intake, including anamnesis, physical examination and in-shoe plantar pressure measurements. Based on clinical reasoning, individual treatment goals were set concerning redistribution of plantar pressure in painful foot regions. Initially, it was considered whether plantar pressure reduction was desirable for the painful region. If this was not the case, for example due to pain avoidance as a result of inflammation, correction or support of foot structures to improve the loading pattern of the foot was considered. Then, a target value for plantar pressure in the painful region was established. Custom-made FOs were designed and manufactured by the podiatrist. These FOs were constructed using prefabricated, semi-rigid supplements with a deep heel cup and contoured medial arch. The supplements were heat-molded to the patient’s foot while using the functional suspension subtalar joint neutral position technique [20, 21]. Based on the findings of the podiatric intake, functional corrections [12–14, 20] (i.e. varus-, valgus corrections, metatarsal bars and metatarsal domes) and shock absorbing padding could be added [13, 20]. The FOs were covered with leather, ethylene vinyl acetate (EVA) or cushioning material. Finally, the achieved plantar pressure redistribution was evaluated by using the feedback of in-shoe plantar pressure measurements. If necessary, the FOs were adapted according to this feedback and the clinical reasoning process of the podiatrist (in one to three rounds).

### Measurements

#### Demographic and clinical characteristics

Sex, age (years), body mass index (kg/m^2^), disease duration (years) and site(s) of foot symptoms as indicated by the patient were recorded. Disease activity was measured using the disease activity score including a 44 joint count (DAS-44; range 0–10) [22]. Joint damage on radiographs in the most affected foot was scored by using the Sharp/ van der Heijde method, including a score for foot joint erosion (range 0–120) and a score for foot joint space narrowing (range 0–48) [23]. The Platto-score was used to quantify forefoot deformity (range 0–12) and rearfoot deformity (range 0–7) in the most affected foot [24]. Radiographs of the feet were scored by a trained physician. All other measurements were performed by two independent clinical research assistants trained in standardized measurements.

#### Foot pain and physical functioning

Foot pain was assessed by using the Foot Function Index (FFI) subscale pain as primary outcome [25], and with an additional Numeric Rating Scale (NRS) for foot pain during walking and during standing. Physical functioning was measured by using FFI subscale disability as primary outcome [25] and an additional 10-m-timed walking test. For this performance-based test, patients were instructed to walk 10 m on a self-selected, comfortable walking pace while wearing their own shoes (without FOs at baseline (T0) and with FOs at follow-up (T1)).

#### Forefoot plantar pressure

Forefoot plantar pressure was expressed as Peak Pressure (PP; the highest pressure measured by a single sensor in the forefoot-region) and Pressure Time Integral (PTI; the integral of peak pressure over time measured in the single sensor showing the PP within the forefoot-region, it reflects the amount of pressure applied to the forefoot-region during the total stance phase) [7]. In-shoe plantar pressure measurements without FOs were assessed at baseline (T0) and with FOs at three months after delivery (follow-up (T1)). The Pedar-X system (Novel GmbH, Munich, Germany) was used to measure in-shoe plantar pressure in the patient’s own shoes at the shoe-sock interface, while walking. After accommodation to the Pedar-X system, a test trial was performed to determine comfortable walking speed. The actual measurement consisted of one trial of walking at a self-selected speed along a 25-m walkway. During all measurements walking speed was monitored and when ≥15% deviant from the test trial, patients were asked to adjust their speed and the trial was repeated [26]. Using Pedar-X Step analysis software (Novel gmbh, Munich, Germany) 30 midgait steps were selected per measurement. Acceleration, deceleration and turning steps were excluded.

### Statistical analysis

Clinical characteristics were described with descriptive statistics. Change scores and effect sizes were calculated for pain, physical functioning and observed forefoot plantar pressure in both the total group and the subgroup. The change (T1-T0) in outcome measures was tested for statistical significance by using paired t-tests. Effect sizes were calculated by using Cohen’s D and were interpreted as 0.2 (small), 0.5 (medium) and 0.8 (large) [27]. For observed forefoot plantar pressure (PP and PTI) the patients’ most painful foot (as indicated by the patient) was included in the analysis. Additionally, estimated differences in forefoot plantar pressure (PP and PTI) were assessed in all measured feet by using multi-level analyses, in which a two-level structure was used (i.e. foot (left/right) clustered within patients) [28]. For the subgroup, generalized estimated equation (GEE) analyses were used to investigate the relationship between change (T1-T0) in forefoot plantar pressure (PTI and PP (independent variables)) and change (T1-T0) in pain or physical functioning as the dependent variables [28]. In both multi-level analysis and GEE analysis, an adjustment was made for plantar pressure at baseline [28]. PASW Statistics 18 software (v.18, SPSS Inc. Chicago, IL, USA) was used to perform the analyses. A significance level of *p* < 0.05 was used in all analyses.

## Results

### Patient characteristics

**Fig. 2:**
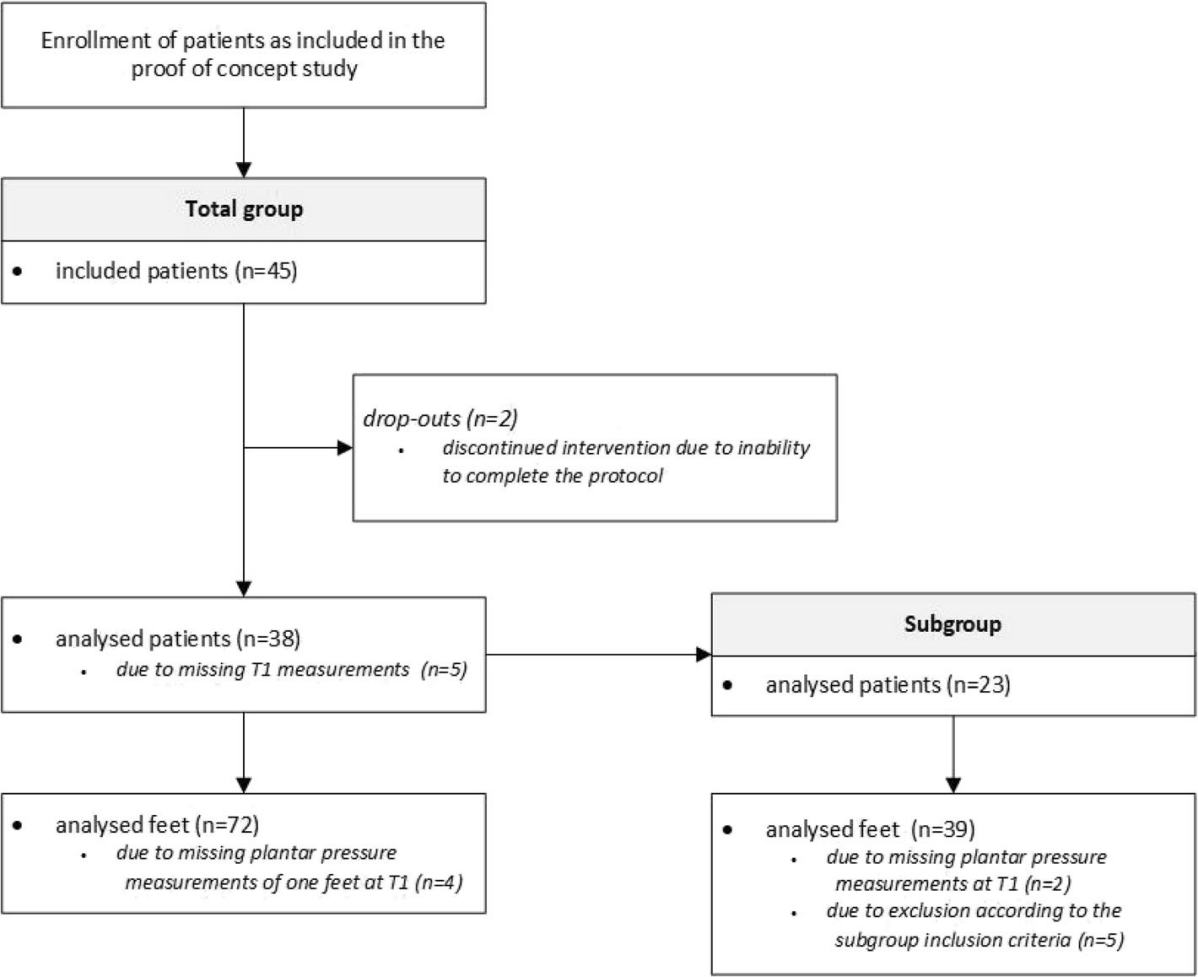
Flow-diagram of patients through the study

Patient flow is depicted in Fig. 2. Forty-five patients were included in the study. Two of the included patients dropped out due to inability to complete the FOs optimization protocol, and in 5 patients T1 measurements were missing, leaving a total group 38 patients for analyses. Out of this group, a subgroup was selected of 23 patients with combined forefoot pain and high plantar pressure (PP ≥ 200 kPa in the central forefoot region (metatarsophalangeal joints 2–3)) at baseline. In the subgroup the relationship between change in forefoot plantar pressure and change in pain and physical functioning was investigated. Table 1 shows the patient characteristics for both groups.

**Table 1:** Patient characteristics

	***Total group (n = 38)***	***Subgroup (n = 23)***
Age, *years*	52.5 (13.9)	53.9 (12.1)
Female, *n (%)*	28 (73.7)	18 (78.3)
Body-mass index, *kg/m2*	26.7 (8.8)	26.3 (4.9)
Disease duration*, *years*	5.5 (1.0;8.5)	6.0 (2.0;10.0)
DAS-44*	1.7 (0.9;2.3)	1.4 (1.0;2.0)
remission (< 1.6), *n (%)*	22 (57.9)	14 (60.9)
low disease activity (1.6–2.3), *n (%)*	9 (23.7)	6 (26.1)
moderate disease activity (2.4–3.6), *n (%)*	5 (13.2)	2 (8.7)
high disease activity (≥ 3.7), *n (%)*	2 (5.3)	1 (4.3)
Location of foot pain, *n (%)*		
rearfoot	2 (5.3)	–
forefoot	29 (76.3)	23 (95.8)
hallux	2 (5.3)	–
combination	5 (13.2)	1 (4.2)
Uni−/ bilateral foot pain, *n (%)*		
unilateral	5 (13.2)	2 (8.7)
bilateral	33 (86.8)	21 (91.3)
Sharp / van der Heijde score feet*		
total score *(range 0–168)*	1.0 (0.0;13.8)	1.0 (0.0;28.0)
foot joint erosion *(range 0–120)*	0.0 (0.0;1.0)	0.0 (0.0;11.0)
joint space narrowing *(range 0–48)*	0.0 (1.0;0.3)	0.00 (0.0;4.0)
Platto-score*		
total score *(range 0–19)*	2.0 (1.0;4.0)	1.8 (1.0;4.4)
forefoot deformity *(range 0–12)*	1.0 (0.0;3.0)	0.8 (0.0;3.0)
rearfoot deformity *(range 0–7)*	1.0 (1.0;1.5)	1.0 (1.0;1.5)
FOs wearing time a day, *n (%)*		
< 1 h	1 (2.9)	0 (0)
1–4 h	8 (22.9)	4 (16.7)
4–8 h	13 (37.1)	10 (41.7)
8–12 ours	8 (22.9)	2 (8.3)
> 12 h	5 (14.3)	3 (12.5)

Values are presented as mean ± SD unless otherwise indicated. * Values are presented as median (IQR). DAS-44 = disease activity score including 44 joints

### Outcomes on pain, physical functioning and forefoot plantar pressure

#### Total group

Table 2 shows the outcomes and effect sizes on pain, physical functioning and forefoot plantar pressure after 3 months of FOs delivery for the total group of 38 patients. Statistically significant improvement on pain and physical functioning were found with, respectively, a medium and large effect size. In-shoe plantar pressure measurements showed a statistically significant PTI reduction (11%) with a small effect size and a non-significant PP reduction (4%) in the patient’s most painful foot (38 ft out of the 38 patients). Similar results were found with the analyses of estimated forefoot plantar pressures in all 72 measured feet (out of the 38 patients).

**Table 2:** Baseline- (T0), follow up- (T1) and change- (Δ T0-T1) scores, and effect sizes for pain, physical functioning and forefoot plantar pressure in the total group (*n* = 38)

***Analysed patients***	**N**	**T0**	**T1**	**Δ T0-T1**	**ES**	***P*-value**
*pain*						
FFI pain (range 0–100), *primary outcome*	38	42.53 (23.92)	28.66 (23.50)	−13.87 (20.00)	0.69	< 0.001
NRS foot pain during walking (range 0–10)	38	5.03 (2.49)	3.37 (2.76)	−1.66 (2.15)	0.77	< 0.001
NRS foot pain during standing (range 0–10)	37	3.95 (2.84)	2.97 (2.70)	−0.97 (2.28)	0.43	0.014
*physical functioning*						
FFI disability (range 0–100), *primary outcome*	38	32.47 (23.38)	22.40 (24.66)	−10.07 (12.25)	0.82	< 0.001
10-m walking time, *seconds*	37	8.78 (1.97)	8.30 (1.33)	−0.49 (1.48)	0.33	0.054
*observed forefoot plantar pressure*						
PP central forefoot (kPa)	38	246.98 (78.22)	236.62 (89.28)	−10.37 (76.62)	0.14	0.40
PTI central forefoot (kPa s)	38	68.84 (28.21)	61.61 (20.27)	−7.23 (20.64)	0.35	0.033
***Analysed feet***	**N**			**Δ T0-T1**		***P-value***
*estimated forefoot plantar pressure*						
PP central forefoot (kPa)	72			−14.20 (10.27)*		0.18
PTI central forefoot (kPa s)	72			−7.07 (2.23)*		0.003

Values are presented as mean ± SD unless otherwise indicated. * Values are presented as estimated mean difference and standard error. *PP* Peak Pressure, *PTI* Pressure Time Integral, *FFI* foot function index, *NRS* numeric rating scale, *ES* effect size

#### Subgroup

The subgroup consisted of 23 patients with both forefoot pain and high plantar pressure (PP ≥ 200 kPa in the central forefoot region (metatarsophalangeal joints 2–3)) at baseline. Table 3 shows the outcomes and effect sizes on pain, physical functioning and forefoot plantar pressures after 3 months of FOs delivery for the subgroup. Statistically significant improvements on pain and physical functioning were found, with a medium effect size. For observed forefoot PP and PTI a statistically significant reduction, respectively 14 and 16%, in the patients’ most painful foot (23 ft out of the 23 patients) with a medium effect size was found. Results were similar for forefoot PP and PTI reduction in all 39 measured feet (out of the 23 patients).

**Table 3:** Baseline- (T0), follow up- (T1) and change- (Δ T0-T1) scores, and effect sizes for pain, physical functioning and forefoot plantar pressure in the subgroup of patients with combined forefoot pain and high plantar pressure at baseline (*n* = 23)

***Analysed patients***	**N**	**T0**	**T1**	**Δ T0-T1**	**ES**	***P*-value**
*pain*						
FFI pain (range 0–100), *primary outcome*	21	34.18 (15.91)	25.23 (19.29)	−8.95 (14.44)	0.62	0.010
NRS foot pain during walking (range 0–10)	21	4.10 (2.53)	2.81 (2.16)	−1.29 (1.95)	0.66	0.007
NRS foot pain during standing (range 0–10)	20	3.15 (2.52)	2.55 (1.99)	−0.60 (1.70)	0.35	0.131
*physical functioning*						
FFI disability (range 0–100), *primary outcome*	21	25.35 (13.88)	17.13 (13.99)	−8.22 (10.38)	0.79	0.002
10-m walking time, *seconds*	22	8.27 (1.08)	8.00 (1.02)	−0.27 (0.77)	0.35	0.110
*observed forefoot plantar pressure*						
PP central forefoot (kPa)	23	288.23 (62.90)	246.85 (66.14)	−41.37 (65.07)	0.64	0.006
PTI central forefoot (kPa s)	23	76.03 (15.01)	63.77 (15.14)	−12.26 (17.83)	0.69	0.003
***Analysed feet***	**N**			**Δ T0-T1**		***P*-value**
*estimated forefoot plantar pressure*						
PP central forefoot (kPa)	39			−28.43 (10.53)*		0.012
PTI central forefoot (kPa s)	39			−12.52 (2.69)*		< 0.001

Values are presented as mean ± SD unless otherwise indicated. * Values are presented as estimated mean difference and standard error. *PP* Peak Pressure, *PTI* Pressure Time Integral, *FFI* foot function index, *NRS* numeric rating scale, *ES* effect size

### Relationship between change in forefoot plantar pressure and change in pain and physical functioning

Table 4 shows the relationship between change in forefoot plantar pressure and change in pain and physical functioning in the subgroup. The effect estimate (*B* in Table 4) reflects the amount of units in which the dependent variable (pain or physical functioning) changes when the independent variable (PP or PTI) changes one unit. No statistically significant associations between change in forefoot plantar pressure and change in pain and physical functioning were found.

**Table 4:** Relation between change in forefoot plantar pressure and change in foot pain and physical functioning (*n* = 23)

	*Δ****Foot pain***						*Δ****Physical functioning***			
	FFI pain		NRS walking		NRS standing		FFI disability		10-m walking time^*^	
	*Β (SE)*	*p-value*	*Β (SE)*	*p-value*	*Β (SE)*	*p-value*	*Β (SE)*	*p-value*	*Β (SE)*	*p-value*
*Δ****Plantar pressure***										
PP / 10 kPa	−2.2 (0.8)	0.79	−0.1 (0.1)	0.58	0.0 (0.1)	0.63	0.3 (0.5)	0.59	0.0 (0.0)	0.70
PTI / 10 kPa s	−3.2 (2.2)	0.15	0.4 (0.3)	0.14	0.2 (0.2)	0.43	−0.5 (1.7)	0.78	0.0 (0.1)	0.85

*FFI* foot function index, *NRS walking* numeric rating scale foot pain during walking, *NRS standing* numeric rating scale foot pain during standing, *PP* peak pressure (kPa), *PTI* Pressure Time Integral (kPa s). * performance-based test

## Discussion

The results of our study showed that wearing FOs, developed according to a FOs optimization protocol by using the feedback of in-shoe plantar pressure measurements, leads to significant improvements on pain and physical functioning, as well as a significant reduction of forefoot plantar pressure. However, there were no statistically significant relationship between change in plantar pressure and changes in pain or physical functioning.

In the present study reduction of pain and improvement of physical functioning, with respectively medium and large effect sizes were found. The outcome on pain is comparable to within-group differences reported in RCTs investigating the effect of FOs, showing pain reduction with medium [20, 29] and large [30, 31] effect sizes. In these studies follow-up ranged from 3 [31] to 30 [20] months, and sample sizes from 24 [31] to 81 [20] patients. Since we studied the results of an optimization protocol we expected to find greater effects on pain reduction. Future research with a head-to-head comparison is needed to demonstrate whether the optimization protocol has an added value over FOs developed without the use of plantar pressure feedback. The results on pain and physical functioning of our subgroup (23 patients with forefoot pain and high plantar pressure) were comparable to results found for the total group (38 patients). Furthermore, results were clinically relevant as the minimal important differences (MID) for FFI pain (12.3 points improvement [32]) and for FFI disability (6.7 points improvement [32]) were reached in the present study. Therefore, wearing FOs developed according to the FOs optimization protocol may lead to clinically relevant improvements in pain and physical functioning.

In the present study a forefoot plantar pressure (PTI) reduction of 11% with a small effect size was found, based on measurements assessed before FOs delivery and after 3 months of wearing FOs. Several studies reported forefoot plantar pressure reduction in RA patients while wearing conventional custom-made FOs compared to a control-condition [12–14, 18, 33, 34]. However, in these studies repeated measures were assessed in a single session and no follow-up results of plantar pressure were reported. Furthermore, forefoot plantar pressure reduction with varying percentages were reported in the literature: PP reduction ranges from 7 to 34% [12–14, 18, 33, 34], and PTI reduction from 12 to 36% [12, 13, 18, 34]. This variation can possibly be explained by different methods for designing FOs or by different baseline characteristics of the studied populations. In a population with higher forefoot plantar pressure at baseline there is a greater potential for reduction, as illustrated by the greater pressure reduction achieved in our subgroup (14% PP reduction and 16% PTI reduction) compared to our total group (4% PP reduction and 11% PTI reduction).

The hypothesis that more plantar pressure reduction leads to more pain reduction and subsequent improvement in physical functioning is not supported by the findings of the present study. Nevertheless, the hypothesis is biologically plausible and forms one of the basic principles for prescribing FOs in patients with RA-related foot problems [12, 18, 20]. A possible explanation for the inability to detect a relationship could be the small sample size of the subgroup. Furthermore, it could be possible that there is a threshold for plantar pressure reduction. Perhaps, plantar pressure reduction up to the threshold-value would lead to relevant improvement on pain and physical functioning outcomes, and additional pressure reduction (over the threshold-value) would not trigger further improvements. This would implicate that focussing on plantar pressure reduction in FOs-treatment is only to a certain level useful. Moreover, reduction of plantar pressure seems to be important in patients with a combination of pain and high pressure in a certain foot region (biomechanical impairment). In these patients FOs designed for off-loading in this foot region seems justified. In cases with relatively low plantar pressure values in the painful foot region (for example due pain avoidance in case of inflammation) another FOs-treatment strategy seems necessary. Likely, the working-mechanism of FOs in patients with RA-related foot problems is based on more components than solely plantar pressure reduction. Probably, the amount in which FOs correct or support foot structures in order to control the position of the feet during weight-bearing and to reduce shearing forces, play an important role in the working mechanism of FOs [35]. Furthermore, a placebo effect is a mechanism that should be considered [29]. To better understand how FOs work in the treatment of RA-related foot problems, larger studies exploring the potential mechanisms underlying the observed effects on pain and physical functioning are warranted.

Although the results of the present study showed no evidence of the supposed relationship between plantar pressure and clinical outcomes, an optimal plantar pressure distribution may contribute to delaying forefoot joint damage and deformities, and prevention of abnormal callosities and wounds on the plantar surface of the foot [7, 35]. Besides the characteristics of the prescribed FOs, compliance and the interaction between FOs and shoes worn by the patient may play an important role in the clinical results of the treatment [35]. Analyses of compliance in the present study showed that only 37% of the included patients wore the FOs more than 8 h a day. Therefore, strategies to improve compliance (targeting usability and acceptance) should be considered [36]. Furthermore, good communication between prescribing clinicians and the individual patients is of great importance [35].

The present study has some strengths and limitations. A strength is the follow-up measurements of in-shoe plantar pressure after 3 months of wearing FOs. Another strength is the mixed model multilevel analyses which enabled us to use different areas of both feet of the same patient, apart from dependency within a person. Therefore, data from both feet of one patient could be used. The following limitations were identified. First, in comparison with the literature the follow-up of 3 months was relatively short for the outcomes pain and physical functioning [15, 16]. Second, due to the relatively small sample size, the statistical power to establish associations of change in pressure with change in outcomes was limited. Third, an individual shoe-advice was given based on the clinical reasoning process of the podiatrist. This advice consisted at least of sufficient room in the toe box and a stiff sole allowing a heel-to-toe gait. Data on the numbers, specific content and degree of follow-up of the individual shoe-advices is lacking. Therefore, analysis of the potential role of the shoe-advise on the outcomes was not possible.

## Conclusions

Foot orthoses developed according to a protocol for improving the plantar pressure redistribution properties lead to medium to large improvements in pain and physical functioning. The hypothesis that more pressure reduction would lead to better clinical outcomes could not be proven.

## Data Availability

The datasets used/or analysed during the current study are available from the corresponding author on a reasonable request.

## Abbreviations

RA: Rheumatoid arthritis
FOs: Foot orthoses
ES: Effect size
EVA: Ethylene vinyl acetate
DAS-44: Disease activity score including a 44 joint count
FFI: Foot Function Index
NRS: Numeric Rating Scale
PP: Peak pressure
PTI: Pressure time integral
GEE analysis: Generalized estimated equation analysis
